# Equine infectious anemia virus Mat protein serves as a Tat cofactor to facilitate viral transcription

**DOI:** 10.1128/jvi.00828-26

**Published:** 2026-07-27

**Authors:** Xiangmin Zhang, Weiwei Ma, Xiaohua Ma, Xing Guo, Weiguo Zhang, Xue-Feng Wang, Xiaojun Wang

**Affiliations:** 1 State Key Laboratory of Animal Disease Control and Prevention, Harbin Veterinary Research Institute of Chinese Academy of Agricultural Sciences 687216, Harbin, China; 2 Institute of Western Agriculture, Chinese Academy of Agricultural Sciences 838119, Changji, China; 3 China-Kazakhstan Joint Laboratory for Herbivorous Animal Disease Research, Harbin Veterinary Research Institute, Chinese Academy of Agricultural Sciences 111613, Harbin, China; Icahn School of Medicine at Mount Sinai, New York, New York, USA

**Keywords:** Tat, transcriptional regulation, lentivirus, EIAV

## Abstract

Equine infectious anemia virus (EIAV) is a member of the *Lentivirus* genus of the *Retroviridae* family and serves as an important model for studying lentiviral pathogenesis. A novel EIAV-encoded protein, designated Mat, was previously identified in our laboratory, yet its biological functions remained unclear. Here, we demonstrate that disruption of Mat moderately reduced EIAV replication in equine monocyte-derived macrophages, and this defect was restored when Mat was supplied in *trans*. Further analyses revealed that Mat exhibits nuclear localization and enhances Tat-mediated transcriptional activation of the long terminal repeat (LTR), an essential and conserved step in lentiviral replication, by interacting with viral Tat and the host factor Cyclin T1 (CycT1), but not with the trans-acting responsive RNA element (TAR) within the LTR. Moreover, we found that Mat stabilizes EIAV Tat (eTat) and promotes the association of eTat with both TAR and equine CycT1. These findings indicate that Mat serves as a cofactor of Tat to regulate EIAV replication.

**IMPORTANCE:**

In lentiviruses, Tat-mediated activation of the LTR promoter is essential for viral replication. Mechanistically, Tat binds to TAR (a structured RNA at the 5′ end of nascent viral transcripts, corresponding to the R region of the LTR), recruits host factors, including CycT1, and facilitates RNA polymerase II elongation, thereby promoting efficient production of full-length viral transcripts required for lentiviral replication. We recently identified an uncharacterized EIAV protein, Mat. Here, we show that Mat inactivation moderately attenuates EIAV replication *in vitro*, and this defect is rescued by ectopic Mat expression. Mat enhances Tat-mediated activation of EIAV LTR activity, and we further confirm that Mat interacts with EIAV Tat (eTat) and equine CycT1 (eqCycT1), stabilizes eTat, and promotes the association of eTat with both TAR and eqCycT1. These results suggest that Mat acts as an eTat cofactor in EIAV transcriptional regulation, providing insights into the complex transcriptional regulatory mechanisms of lentiviruses.

## INTRODUCTION

Equine infectious anemia virus (EIAV), a member of the *Retroviridae* family, is a lentivirus that causes persistent infection in equids (1). EIAV infection causes characteristic clinical symptoms, such as fever, anemia, weight loss, and depression, and can lead to death in severe cases (2). Similar to human immunodeficiency virus type 1 (HIV-1), the EIAV replication cycle is initiated by entry into target cells and the release of its RNA into the cytoplasm (3). Then, the EIAV single-stranded RNA genome is reverse transcribed to double-stranded DNA (dsDNA), catalyzed by the viral reverse transcriptase. Subsequently, the viral dsDNA is transported into the nucleus and integrated into the host genome by the activity of the viral integrase. The integrated proviral DNA then serves as a template for viral transcription, which represents an essential step for viral replication (4).

The 5′ long terminal repeat (LTR) of lentiviruses functions as a promoter that drives viral transcription. This process is cooperatively regulated by host RNA polymerase II (Pol II) and the viral Tat protein (5). Without Tat, the LTR promoter exhibits only basal transcriptional activity. Pol II initiates transcription, but elongation is inefficient, resulting in short, abortive transcripts (6–8). As a key transactivator, lentiviral Tat binds to the transactivation response element (TAR), a structured RNA at the 5′ end of nascent transcripts corresponding to the LTR R region, and recruits positive transcription elongation factor b (P-TEFb) (9, 10). Through its interactions with both TAR and P-TEFb, Tat directly increases the transcriptional elongation capacity of Pol II, enabling the synthesis of high levels of full-length, functional viral RNAs, in a manner essential for lentiviral replication (11). Cyclin T1 (CycT1), a regulatory subunit of P-TEFb, acts as a molecular bridge to stabilize the association between Tat and P-TEFb, while facilitating the assembly of the transcription machinery at the viral promoter (12, 13). EIAV Tat protein (eTat), comprising 75 amino acids, exhibits a relatively simplified domain compared to primate lentiviruses such as HIV-1 and Simian immunodeficiency virus (14, 15). Previous studies have comprehensively elucidated the mechanism by which eTat mediates EIAV LTR promoter activation, confirming that this process is strictly dependent on equine CycT1 (eqCycT1) (16, 17). However, human CycT1 (huCycT1) fails to support EIAV Tat-driven LTR transcriptional activity, highlighting a species-specific functional requirement for CycT1 in EIAV replication (13, 18).

Lentiviral accessory proteins are crucial for modulating viral replication (4, 19). Although extensive research has characterized interactions between host proteins and lentiviral regulatory or structural proteins, the functional significance of viral protein-protein interactions remains less thoroughly explored. Previous studies have reported that HIV-1 accessory proteins can regulate transcriptional activity by interacting with HIV-1 Tat (hTat) (20). For instance, several studies have demonstrated that HIV-1 Vpr and Nef interact functionally with hTat to enhance HIV-1 LTR promoter activity, thereby promoting viral gene expression (21, 22). In contrast, interactions between HIV-1 NC or Rev and hTat lead to the degradation of Tat through the proteasomal pathway, ultimately inhibiting hTat functions (23, 24). Despite these insights in HIV-1, potential interactions between eTat and accessory proteins in EIAV remain unexplored.

Mat is a novel EIAV-encoded accessory protein identified in our laboratory. The gene is designated *mat* because its first exon overlaps with the matrix-coding domain of *gag* and its second exon overlaps with the transmembrane-coding domain of *env* (25). Its biological function has remained uncharacterized. In this paper, we demonstrate that the deficit of Mat moderately attenuated EIAV replication ability, and the replication defect of Mat-deficient EIAV was moderately rescued by trans-expression of Mat. Furthermore, our results showed that Mat functions as an eTat coactivator to facilitate eTat-mediated LTR activity.

## RESULTS

### Inactivation of Mat attenuated the replication of EIAV

**Fig 1:**
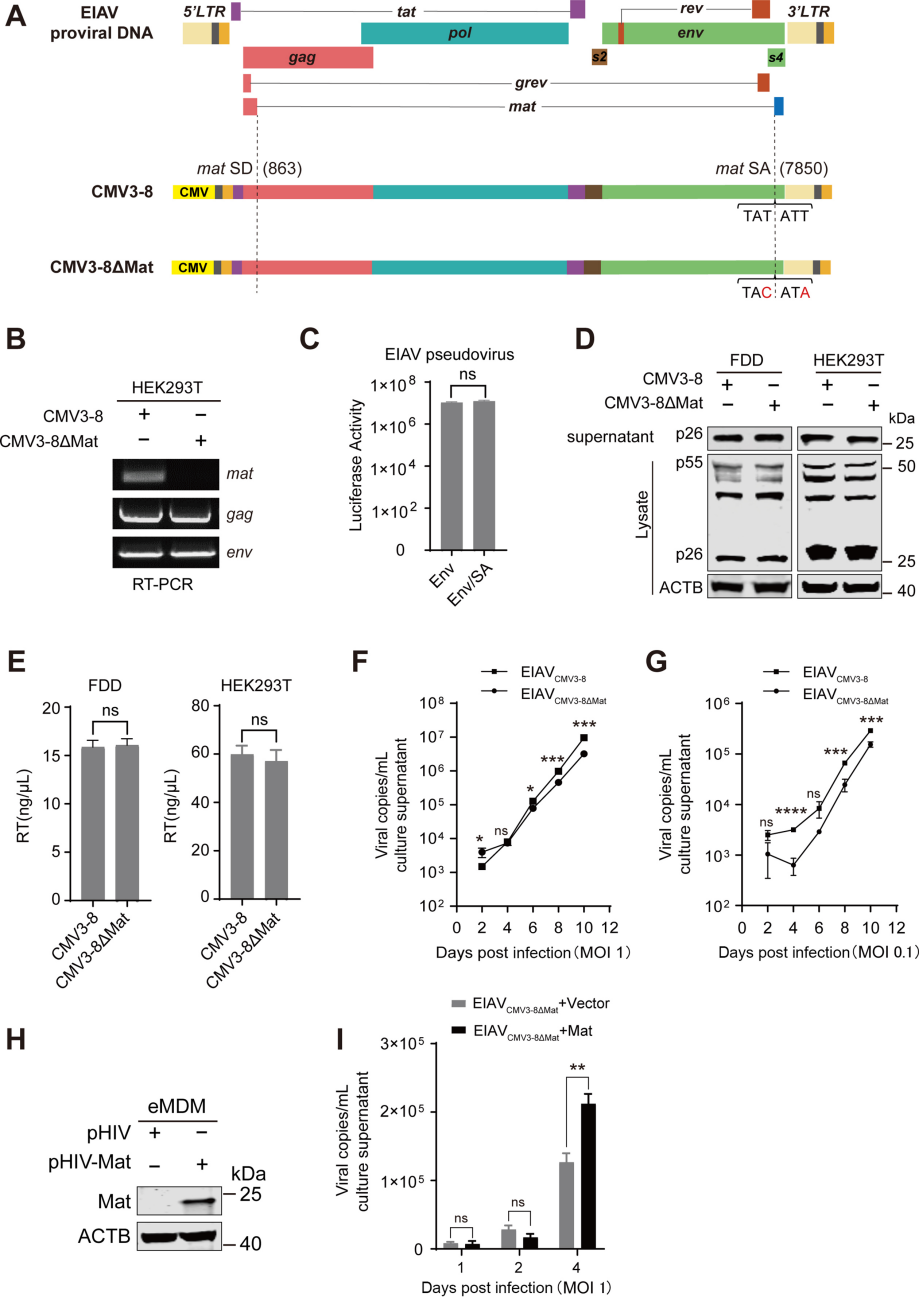
The defect of Mat attenuated the replication of EIAV. (**A**) Schematic representation of CMV3-8ΔMat. The EIAV genome is depicted above, with the CMV-promoter-driven EIAV infectious clone shown below. CMV3-8 represents the wild-type EIAV infectious clone. CMV3-8ΔMat represents the *mat* mRNA-defective EIAV infectious clone. SD, splice donor site; SA, splice acceptor site. The numbers indicate the genomic nucleotide positions of the splice sites within the *mat* mRNA. The mutated nucleotides are highlighted in red. (**B**) RT-PCR analysis was performed on RNA isolated from HEK293T cells transfected with CMV3-8 or CMV3-8ΔMat, using specific primers to detect *mat*, *env*, and *gag* mRNAs, respectively. (**C**) Env codon mutations corresponding to the mat SA have no impact on EIAV entry. EIAV luciferase reporter viruses with a wild-type Env or an Env mutant (carrying two nucleotide mutations corresponding to the SA of mat mRNA) normalized by reverse transcriptase (RT) activity were inoculated into HEK293T/ELR1 cells. At 24 h post-transfection (hpt), the cells were lysed, and luciferase activity was measured for each group. Data are presented as the mean ± SD of three independent experiments. ns, not significant (*P* > 0.05). (**D**) FDD and HEK293T cells were transfected with equivalent amounts (1 μg) of CMV3-8 or CMV3-8∆Mat. At 48 hpt, culture supernatants were ultracentrifuged to concentrate the viral particles. Viral capsid protein (CA, P26) in the supernatant and Gag protein in the cell lysates were analyzed by Western blotting using an anti-p26 antibody. Data shown are representative of three independent experiments. (**E**) Experiments were performed as described in panel D. Supernatants were harvested, and RT activity was measured. Data are presented as the mean ± SD of three independent experiments. ns, not significant (*P* > 0.05). (**F and G**) Kinetics of EIAV_CMV3-8WT_ and the EIAV_CMV3-8∆Mat_ replication. eMDMs were inoculated with equivalent titers of EIAV_CMV3-8_ or EIAV_CMV3-8∆Mat_ at an MOI of 1 (**F**) or 0.1 (**G**). At the indicated time points, viral RNA was extracted from the supernatants and quantified using RT-qPCR. Data are shown as mean ± SD of three technical replicates from a representative experiment. The experiment was repeated three times independently with similar results. ns, not significant (*P* > 0.05); *P < 0.05; ***P < 0.001; and ****P < 0.0001. (**H**) Detection of Mat expression in eMDMs infected with Mat-expressing pseudotyped HIV. eMDMs were infected with the indicated pseudotyped HIV (pHIV-Mat-Flag or control pHIV) and harvested 48 h post-infection. Mat expression was analyzed by Western blotting using an anti-Flag antibody. Data shown are representative of three independent experiments. (**I**) Replication of Mat-deficient EIAV in eMDMs is rescued by trans-complementation with Mat. EIAV_CMV3-8∆Mat_ (MOI of 1) with pseudotyped HIV-1 either containing Mat-Flag or without Mat-Flag (as a negative control) was inoculated into eMDMs. Viral RNA was extracted from the supernatants at the indicated time points and quantified using RT-qPCR. Data are shown as mean ± SD of three technical replicates from a representative experiment. The experiment was repeated two times independently with similar results. ns, not significant (*P* > 0.05); **P < 0.01.

We previously identified a novel EIAV protein, Mat; however, its biological function has not been characterized. In this study, we aimed to investigate its role in EIAV replication. Mat is a small protein composed of 148 amino acids, and its encoding gene, *mat*, contains two exons. Notably, the coding region of *mat* partially overlaps with the *gag* and *env* genes. This overlap prevents the use of traditional genetic manipulation strategies, such as frameshift or deletion mutations, to abolish Mat expression in the context of the EIAV infectious clone. To generate a Mat-deficient EIAV infectious clone, we used a cytomegalovirus (CMV) promoter-driven EIAV infectious clone (CMV3-8) as a template and introduced two nucleotide synonymous mutations near the splice acceptor (SA) site of *mat* (T7850C and T7853A) within the EIAV genome (GenBank GU385361.1) to disrupt splicing of the *mat* transcript. The resulting construct was designated CMV3-8ΔMat (Fig. 1A). To confirm successful *mat* gene disruption, reverse transcription-PCR (RT-PCR) was performed to detect *mat* transcripts in HEK293T cells transfected with either CMV3-8 or CMV3-8ΔMat, with *gag* and *env* transcripts detection as controls. As predicted, CMV3-8ΔMat-transfected cells showed a complete absence of *mat* mRNA, whereas *gag* and *env* mRNA levels were comparable between CMV3-8 and CMV3-8ΔMat-transfected cells (Fig. 1B). These results indicate that *mat* transcript splicing is specifically disrupted in CMV3-8ΔMat, without affecting the expression of other viral mRNAs. Given that the introduced mutations are located within the *env* coding sequence, we further confirmed that these mutations do not affect the ability of Env to mediate viral entry (Fig. 1C).

Next, the equivalent amount of either CMV3-8 or CMV3-8ΔMat expression plasmid was transfected into fetal donkey dermal (FDD) cells, which are permissive for the replication of EIAV, and HEK293T cells. The amounts of virion-associated p26 in the culture supernatant and Gag protein (p55) in the cell lysate were assessed at 48 h post-transfection (hpt) using Western blotting (WB). The results showed no significant differences in viral protein expression levels either in the supernatant or the cell lysate (Fig. 1D). We also attempted to detect the Mat protein in CMV3-8 and CMV3-8ΔMat using the previously generated Mat-specific monoclonal antibodies (26). However, despite employing multiple experimental approaches, we were unable to reliably detect Mat (Fig. S1), likely due to its low endogenous expression levels below the detection threshold of our assay. Nevertheless, the specific disruption of the *mat* transcript (Fig. 1B) provides genetic evidence for the loss of Mat function in the CMV3-8ΔMat construct. In addition, we quantified the amounts of virions in the supernatants from CMV3-8- and CMV3-8ΔMat-transfected cells using a commercial reverse transcriptase activity (RT) assay kit. No differences in RT activity were found between viruses produced by the two constructs in both HEK293T and FDD cells, although baseline RT activity was lower in FDD cells than in HEK293T cells, likely due to lower transfection efficiency (Fig. 1E). The above results demonstrate that the absence of Mat does not affect viral particle production and release in the context of CMV-driven EIAV molecular clones.

To assess the impact of Mat inactivation on EIAV replication, equine monocyte-derived macrophages (eMDMs, the primary target cells of EIAV) were infected with equal titers of EIAV_CMV3-8_ and EIAV_CMV3-8ΔMat_. Viral growth kinetics over the course of infection revealed that Mat deficiency consistently attenuated the replication capacity of EIAV at both a multiplicity of infection (MOI) of 1 (Fig. 1F) and an MOI of 0.1 (Fig. 1G). When Mat was expressed in *trans* in infected eMDMs using pseudotyped HIV, it moderately rescued the replication of Mat-deficient EIAV (Fig. 1H and I). Notably, whereas Mat deficiency had no effect on viral expression and release in the transfection assay (Fig. 1D and E), a marked difference in viral replication was observed in the infection assay (Fig. 1F and G). This discrepancy may be attributed to the distinct replication modes employed in each assay: the former involves CMV-driven, single-round viral production, restricted to viral protein synthesis and virion release; in contrast, the latter encompasses the entire viral life cycle, involving the full cascade of LTR-driven, multi-round viral replication cycles. Taken together, these findings suggest that Mat plays a positive regulatory role in EIAV replication.

### Mat facilitates eTat-mediated EIAV LTR transcriptional activity

**Fig 2:**
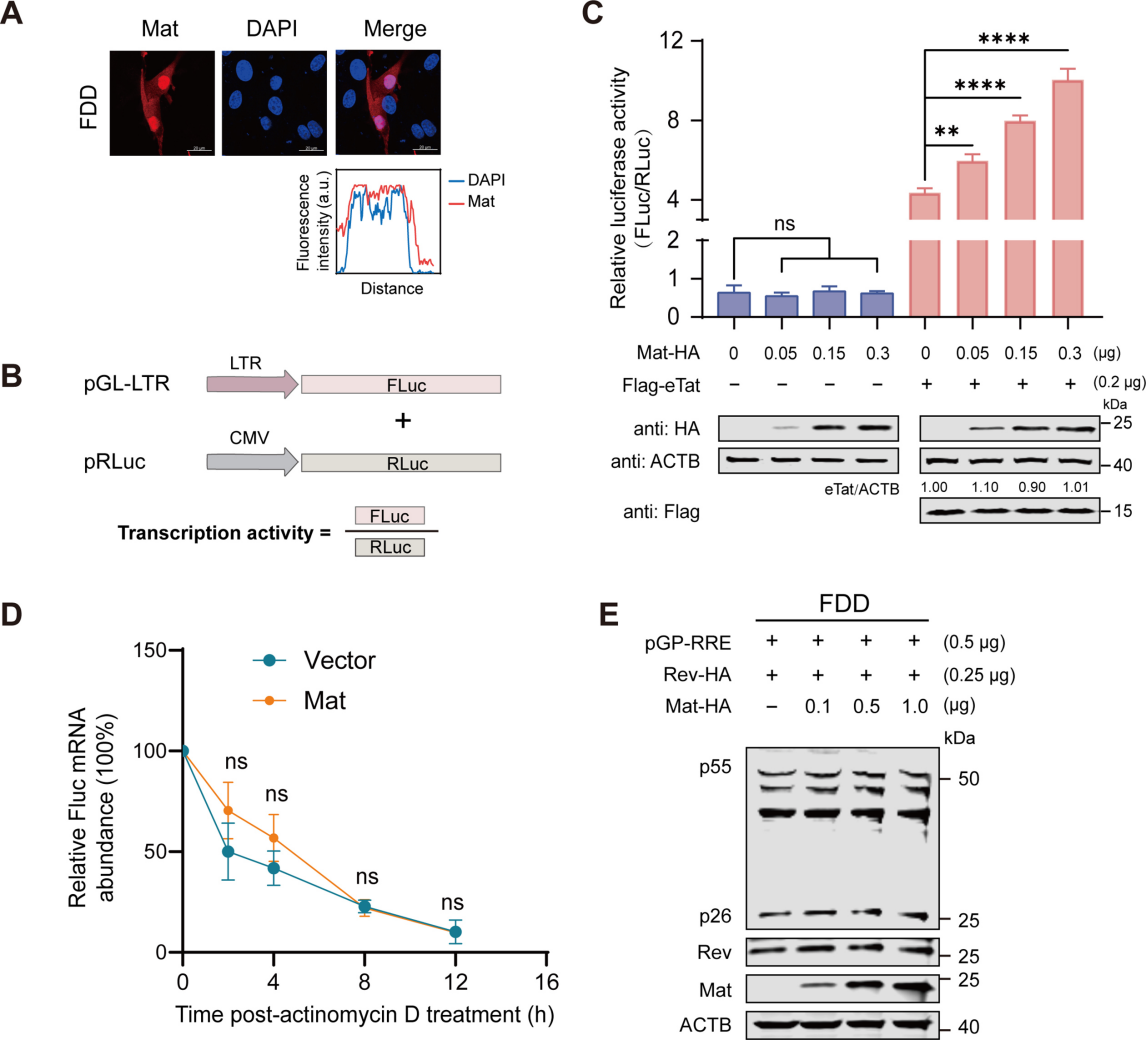
Mat enhanced eTat-mediated LTR activity. (**A**) The localization of ectopically expressed Mat protein in FDD cells. pcDNA-Mat-HA was transfected into FDD cells, and at 24 hpt, cells were fixed, permeabilized, and immunostained with rabbit anti-HA (red) followed by Alexa Fluor 647-conjugated goat anti-rabbit antibodies. Nuclei were counterstained with DAPI (blue) and then observed using confocal microscopy. Scale bar, 20 μm. Representative images of 20 cells are shown. Fluorescence intensity profile of the merged image was analyzed by ZEISS ZEN Blue software. The colored curves represent the fluorescence signals of Mat and DAPI, and their overlapping peaks indicate colocalization of the two signals. (**B**) Schematic diagram of the dual-luciferase reporter assay for detecting EIAV LTR transcriptional activity. The ratio of firefly luciferase (FLuc) to Renilla luciferase (RLuc) represents the relative EIAV LTR transcriptional activity. (**C**) FDD cells were cotransfected with pGL3-LTR and the indicated plasmids. The Renilla luciferase expression plasmid pRL-CMV was cotransfected to normalize transfection efficiency. An empty vector pcDNA3.1(+) was added to normalize the DNA amounts of transfection. The relative luciferase activities were measured to assess the EIAV LTR transcriptional activity. Results represent the means ± SD of three replicate wells from a representative experiment. Independent experiments were performed three times. ns, not significant (*P* > 0.05); **P < 0.01; and ****P < 0.0001. (**D**) The stability of Fluc mRNA. FDD cells were cotransfected with the pGL3-LTR, pCAGGS-Flag-eTat, and either pcDNA-Mat-HA or empty vector. At 24 hpt, cells were treated with actinomycin D (10 μg/mL) to block transcription, and Fluc mRNA levels were detected by RT-qPCR. The Fluc mRNA level at 0 h post-treatment was set as 100%, and the remaining mRNA at each time point was expressed as a percentage of the 0-h value. Data are shown as mean ± SD of three technical replicates from a representative experiment. The experiment was repeated three times independently with similar results. ns, not significant (*P* > 0.05). (**E**) Mat had no effect on the nuclear export of EIAV Rev-mediated mRNAs. The Gag-Pol expression plasmid pGP-RRE was cotransfected with 0.25 μg of EIAV Rev expression plasmid and increasing doses of Mat expression plasmid into FDD cells. At 48 hpt, the cells were harvested and analyzed using WB. Data shown are representative of three independent experiments.

Subcellular localization of a protein is commonly associated with its function and biological role (27, 28). Confocal microscopy analysis revealed that Mat is distributed in both the nucleus and cytoplasm of FDD cells, with predominant nuclear distribution (Fig. 2A). During lentiviral replication, two critical steps occur within the nucleus: proviral transcription and the nuclear export of viral mRNAs. These two steps are key determinants of viral gene expression efficiency and viral replication in lentiviruses, including HIV-1 and EIAV (29). Among them, Tat activates the LTR promoter to drive viral transcription, and Rev mediates the nuclear export of unspliced and incompletely spliced viral mRNAs and their subsequent expression (30–32). To investigate whether Mat plays a role in EIAV transcription, we cotransfected FDD cells with an EIAV LTR-driven luciferase reporter plasmid and increasing amounts of Mat expression plasmid in the presence or absence of an eTat expression plasmid (Fig. 2B). The results showed that Mat alone had no effect on EIAV LTR promoter activity without eTat. However, while eTat markedly increased LTR activity, Mat further enhanced this activity in a dose-dependent manner (Fig. 2C). To determine whether Mat enhances LTR activity by stabilizing LTR-derived mRNAs, we assessed Fluc mRNA stability following transcriptional inhibition with actinomycin D (10 μg/mL) and found that Mat showed no effect on Fluc mRNA stability (Fig. 2D). Collectively, these results suggest that Mat positively regulates EIAV transcription. Next, we investigated whether Mat influences Rev-mediated mRNA nuclear export. To this end, we used a Rev-dependent EIAV Gag-Pol expression system and assessed Gag-Pol protein levels by WB. Our results revealed that Mat at different doses did not alter Gag-Pol protein levels, indicating that Mat does not regulate the Rev-mediated nuclear export of viral mRNA (Fig. 2E). Taken together, these results demonstrate that Mat specifically enhances eTat-mediated LTR transcriptional activity, without affecting LTR-derived mRNA stability or Rev-mediated nuclear export. This finding aligns with the results in Fig. 1, given that Mat specifically enhances LTR-driven transcription, it does not affect viral expression and release in CMV-driven infectious clone assays (independent of LTR-mediated transcription) but modulates viral replication in LTR-dependent infection assays (Fig. 1D through G).

### Mat interacts with eTat

**Fig 3:**
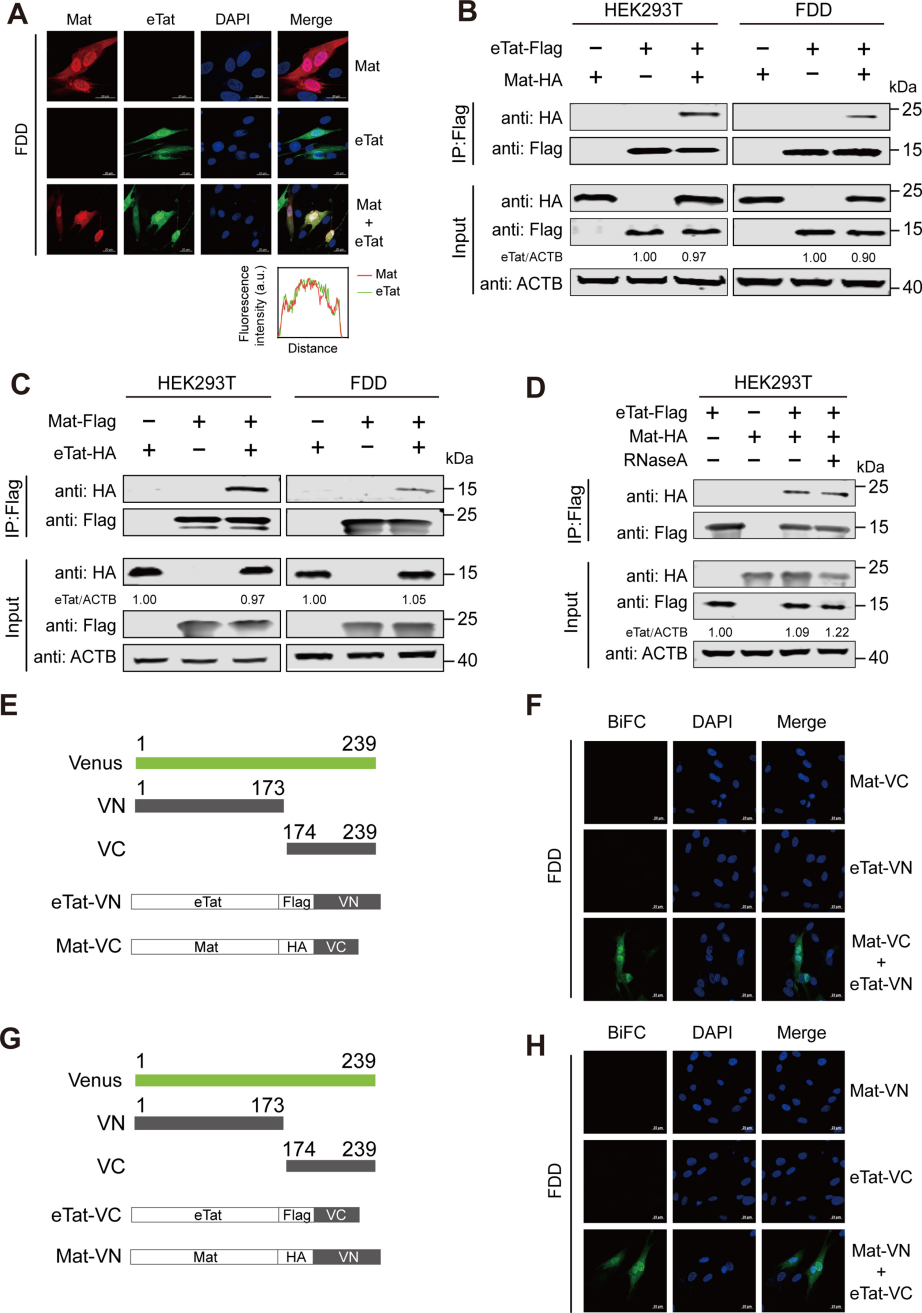
Mat interacts with eTat. (**A**) Mat co-localized with eTat. Mat-HA and eTat-Flag expression plasmids were transfected individually or together into FDD cells. At 24 hpt, cells were fixed, permeabilized, and immunostained with rabbit anti-HA (red) and mouse anti-Flag (green) antibodies followed by Alexa Fluor 647-conjugated goat anti-rabbit antibodies and Alexa Fluor 488-conjugated goat anti-mouse antibodies. Nuclei were counterstained with DAPI (blue) and then observed using confocal microscopy. Scale bar, 20 μm. Representative images of 20 cells are shown. Fluorescence intensity profile of the merged image was analyzed by ZEISS ZEN Blue software. The colored curves represent the fluorescence signals of Mat and eTat, and their overlapping peaks indicate colocalization of the two signals. (**B and C**) Co-IP analysis of Mat and eTat. HEK293T and FDD cells were cotransfected with VR-eTat-Flag and pcDNA-Mat-HA (**B**) or pcDNA-Mat-Flag and VR-eTat-HA (**C**), either individually or together. Cell lysates were subjected to immunoprecipitation using anti-Flag M2 magnetic beads, followed by WB analysis. Data are representative of three independent experiments. (**D**) eTat and Mat interact independently of RNA. HEK293T cells were transfected with the indicated plasmids. Cell lysates were treated with or without RNase prior to IP, then subjected to WB analysis. Data shown are representative of three independent experiments. (**E–H**) BiFC assay for detecting the interaction between Mat and eTat. (**E and G**) Schematic diagrams of two fusion protein combinations: eTat-VN + Mat-VC (**E**) and eTat-VC + Mat-VN (**G**). Mat-HA-VC and eTat-Flag-VN (**F**) and Mat-HA-VN and eTat-Flag-VC (**H**) were expressed individually or together in FDD cells. At 24 hpt, cells were fixed, permeabilized, and visualized using confocal microscopy. Scale bar, 20 μm. Representative images of 20 cells are shown.

To investigate whether Mat functionally associates with eTat, we first examined their subcellular localization by confocal microscopy. Co-expression of Mat-HA and eTat-Flag in FDD cells revealed substantial overlap of their fluorescence signals, particularly in the nuclear region (Fig. 3A). Next, we performed co-immunoprecipitation (Co-IP) assays to test whether Mat interacts with eTat. In both HEK293T and FDD cells, when eTat-Flag was immunoprecipitated using anti-Flag beads, Mat-HA was specifically detected in the precipitate only when both proteins were co-expressed (Fig. 3B). Conversely, when Mat-Flag was used as bait, eTat-HA was similarly co-immunoprecipitated (Fig. 3C), confirming a specific interaction. Considering that lentiviral Tat is an RNA-binding protein, we performed RNase treatment in the Co-IP assay to exclude the possibility that the interaction between Mat and eTat is mediated by RNA. The interaction remained unaffected by RNase treatment, indicating that the association between Mat and eTat is RNA-independent (Fig. 3D). To further test this interaction, we performed a bimolecular fluorescence complementation (BiFC) assay using the constructs illustrated in Fig. 3E and G. Fluorescence was observed in FDD cells co-expressing Mat-VN and eTat-VC (or Mat-VC and eTat-VN), especially within the nucleus, but not when either construct was expressed alone (Fig. 3F and H). Taken together, these results consistently demonstrate that Mat specifically interacts with eTat, and this interaction is RNA-independent.

### The first exon of Mat is the key region enhancing eTat-mediated LTR activity

**Fig 4:**
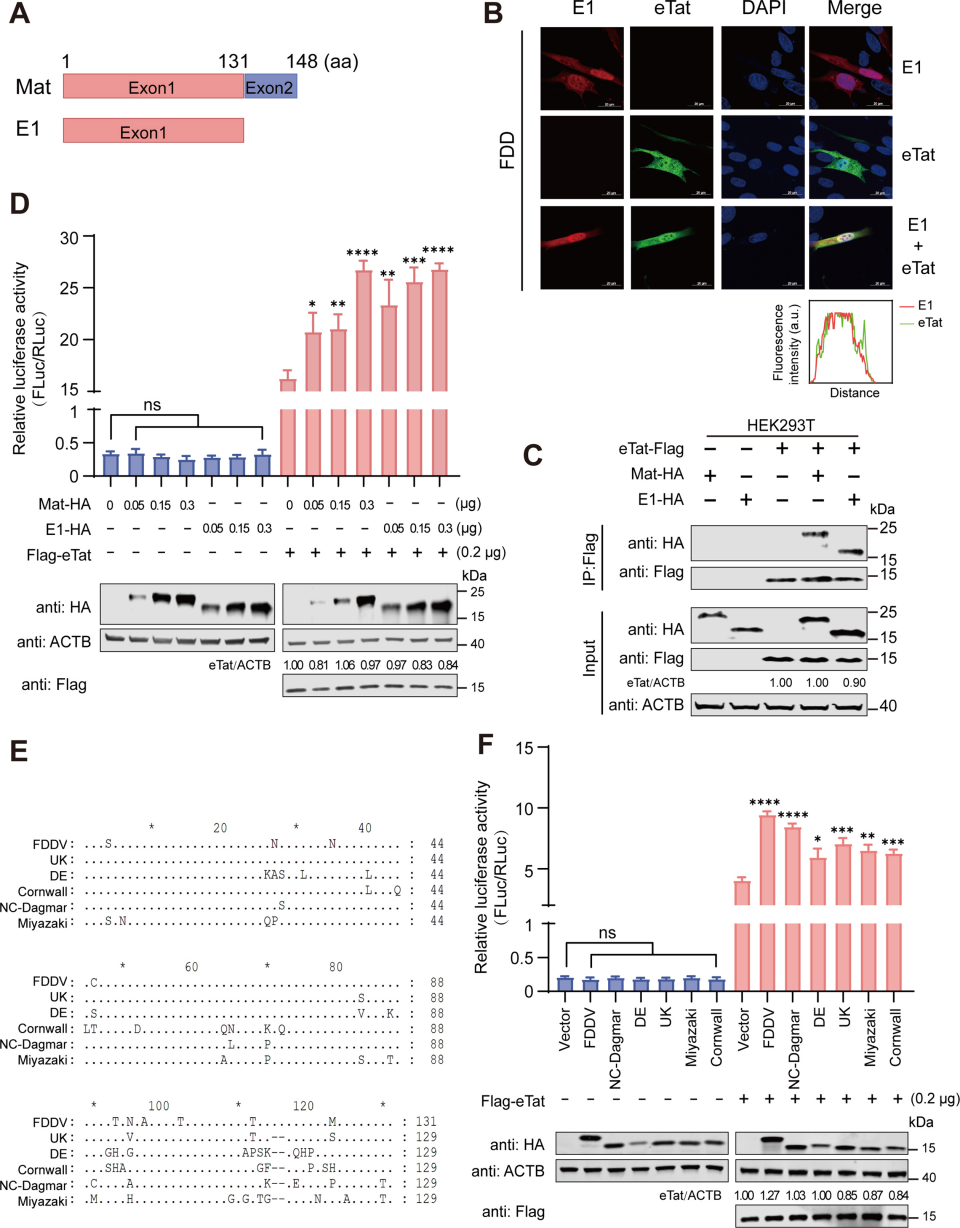
The first exon of Mat is the key region enhancing eTat-mediated LTR activity. (**A**) Schematic diagram of Mat and its Exon 1 (E1) protein. Mat consists of two exons encoding a total of 148 amino acids (aa), with the first exon (here named E1) containing 131 aa. (**B**) E1 co-localized with eTat. E1-HA and eTat-Flag expression plasmids were transfected individually or together into FDD cells. At 24 hpt, cells were fixed, permeabilized, and immunostained with rabbit anti-HA (red) and mouse anti-Flag (green) antibodies followed by Alexa Fluor 647-conjugated goat anti-rabbit antibodies and Alexa Fluor 488-conjugated goat anti-mouse antibodies. Nuclei were counterstained with DAPI (blue) and then observed using confocal microscopy. Scale bar, 20 μm. Representative images of 20 cells are shown. Fluorescence intensity profile of the merged image was analyzed by ZEISS ZEN Blue software. The colored curves represent the fluorescence signals of E1 and eTat, and their overlapping peaks indicate colocalization of the two signals. (**C**) Co-IP analysis of E1 and eTat. HEK293T cells were cotransfected with plasmids expressing eTat-Flag in the presence of E1-HA, Mat-HA, or an empty vector, subjected to IP using anti-Flag M2 magnetic beads, followed by WB analysis. Data shown are representative of three independent experiments. (**D**) E1 enhances eTat-mediated LTR activity. pGL3-LTR was cotransfected with the indicated plasmids into FDD cells. pRL-CMV was cotransfected to normalize transfection efficiency. The relative luciferase activities were measured to assess the EIAV LTR transcriptional activity. Results represent the means ± SD of three replicate wells from a representative experiment. Independent experiments were performed two times. ns, not significant (*P* > 0.05); *P < 0.05; **P < 0.01; ***P < 0.001; and ****P < 0.0001. (**E**) Amino acid sequence alignment of E1 across different EIAV strains. (**F**) E1 of different EIAV strains enhanced eTat-mediated LTR transcriptional activity, as described for panel D.

The Mat protein consists of 148 amino acids encoded by two exons, with the second exon encoding only 17 amino acids (Fig. 4A). To map its functional domains, we constructed an expression vector encoding Mat Exon 1 (E1). Confocal microscopy showed that E1 co-localized with eTat in FDD cells, especially within the nucleus (Fig. 4B). Co-IP assay in HEK293T cells demonstrated the interaction of E1 and eTat (Fig. 4C). Dual-luciferase reporter assay showed that E1 enhanced eTat-mediated EIAV LTR activity, similar to full-length Mat (Fig. 4D). To test whether this function is conserved across different EIAV strains, we aligned the E1 sequences from six representative strains (Fig. 4E) and synthesized the corresponding E1 variants. All six variants enhanced eTat-mediated LTR activity, indicating that this function is conserved despite sequence divergence (Fig. 4F). These results indicate that E1 interacts with eTat and is the key functional region of Mat responsible for enhancing eTat-mediated LTR activity.

### The critical domain of Mat for eTat binding

**Fig 5:**
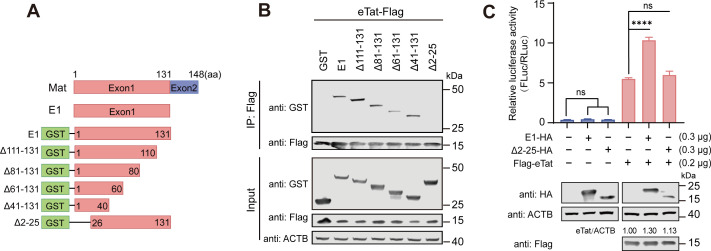
The critical domain of Mat binds eTat. (**A**) Schematic diagram of GST-tagged E1 truncations. (**B**) Co-IP analysis of eTat and GST-E1 truncations. HEK293T cells were cotransfected with VR-eTat-Flag and GST-E1 truncation mutants, subjected to IP using anti-Flag M2 magnetic beads, followed by WB analysis. Data shown are representative of three independent experiments. (**C**) E1Δ2-25 failed to enhance eTat-mediated LTR activity. pGL3-LTR was cotransfected with the indicated plasmids into FDD cells. pRL-CMV was cotransfected to normalize transfection efficiency. The relative luciferase activities were measured to assess EIAV LTR transcriptional activity. Results represent the means ± SD of three replicate wells from a representative experiment. Independent experiments were performed three times. ns, not significant (*P* > 0.05); ****P < 0.0001.

After mapping the functional interaction of Mat and eTat to the E1 domain, we next attempted to identify the minimal region required for this interaction. To this end, we generated a series of GST-tagged E1 deletion mutants and performed binding assays (Fig. 5A). Co-IP analysis showed that the N-terminal 24-amino-acid region of Mat (mutant E1Δ2-25) is essential for binding to eTat (Fig. 5B). Consistent with this, E1Δ2-25 failed to enhance eTat-mediated LTR activity in the dual-luciferase reporter assay (Fig. 5C). Furthermore, confocal microscopy showed that E1Δ2-25 exhibited comparable subcellular localization to E1 (Fig. S2). These results indicate that the impaired binding and functional deficiency of this mutant are not attributed to altered subcellular localization, ruling out abnormal co-localization as a potential possibility for disrupted Mat-eTat interaction.

### Mat interacts with eqCycT1

**Fig 6:**
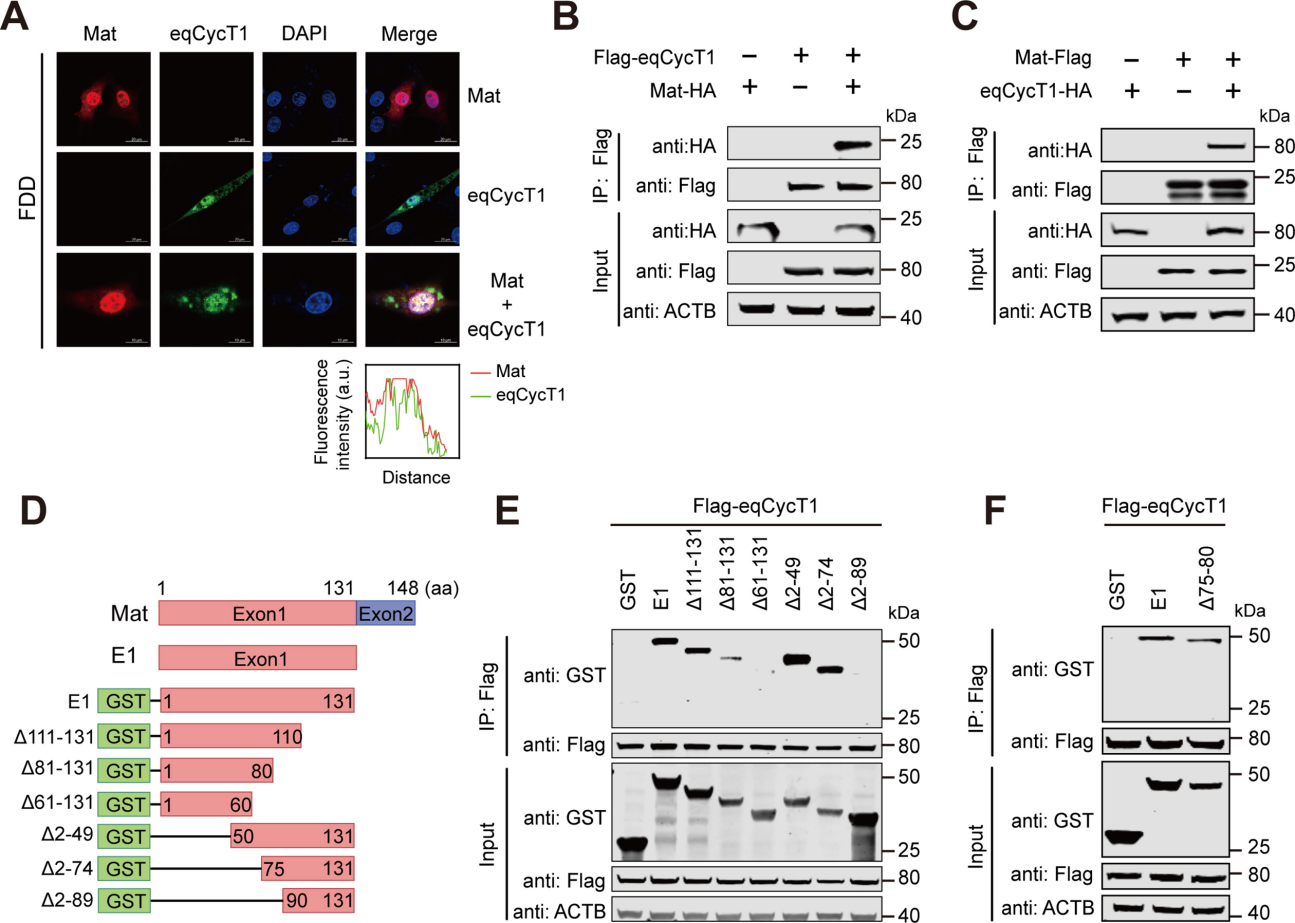
Mat interacts with eqCycT1 and the key domain mapping. (**A**) Mat co-localized with eqCycT1. Mat-HA and Flag-eqCycT1 expression plasmids were transfected individually or together into FDD cells. At 24 hpt, cells were fixed, permeabilized, and immunostained with rabbit anti-HA (red) and mouse anti-Flag (green) antibodies followed by Alexa Fluor 647-conjugated goat anti-rabbit antibodies and Alexa Fluor 488-conjugated goat anti-mouse antibodies. Nuclei were counterstained with DAPI (blue). Scale bar, 20 μm. Representative images of 20 cells are shown. Fluorescence intensity profile of the merged image was analyzed by ZEISS ZEN Blue software. The colored curves represent the fluorescence signals of Mat and eqCycT1, and their overlapping peaks indicate colocalization of the two signals. (**B and C**) Co-IP analysis of Mat and eqCycT1. HEK293T cells were cotransfected with pCAGGS-Flag-eqCycT1 and pcDNA-Mat-HA (**B**) or pcDNA-Mat-Flag and pCAGGS-eqCycT1-HA (**C**), either individually or together. Cell lysates were subjected to IP using anti-Flag M2 magnetic beads, followed by WB analysis. Data are representative of three independent experiments. (**D**) Schematic diagram of GST-tagged E1 truncations. (**E**) Mapping the key domain of Mat that interacts with eqCycT1. HEK293T cells were cotransfected with pCAGGS-Flag-eqCycT1 and GST-E1 truncations, subjected to IP using anti-Flag M2 magnetic beads, followed by WB analysis. Data shown are representative of three independent experiments. (**F**) Co-IP analysis of GST-E1Δ75-80 with eqCycT1. HEK293T cells were cotransfected with indicated plasmids, subjected to IP using anti-Flag M2 magnetic beads, followed by WB analysis. Data shown are representative of three independent experiments.

Since Tat-mediated LTR activity requires the assembly of Tat, eqCycT1, and the viral LTR TAR element into a functional complex, and our data indicate that Mat can enhance Tat-mediated LTR activity, we next investigated whether Mat interacts with eqCycT1. Confocal microscopy showed that Mat co-localized with eqCycT1 in the nucleus of FDD cells (Fig. 6A). Furthermore, reciprocal Co-IP assays revealed that Mat interacted with eqCycT1: Flag-eqCycT1 co-precipitated Mat-HA (Fig. 6B), and conversely, Mat-Flag co-precipitated eqCycT1-HA (Fig. 6C). To map the essential Mat domain that binds to eqCycT1, we generated a series of truncation mutants based on the GST-E1 expression vector (Fig. 6D). The Co-IP results showed that neither E1Δ61-131 nor E1Δ2-89 was able to bind eqCycT1, whereas both E1Δ81-131 and E1Δ2-74 retained this binding capacity (Fig. 6E). To further pinpoint the critical region, we generated an internal deletion mutant E1Δ75-80. Notably, this mutant still retained the ability to interact with eqCycT1 (Fig. 6F). These collective findings suggest that the Mat-eqCycT1 interaction may depend on the overall spatial conformation of Mat rather than on a single linear domain.

### Mat maintains eTat stability and promotes the association of eTat with its transcriptional partners

**Fig 7:**
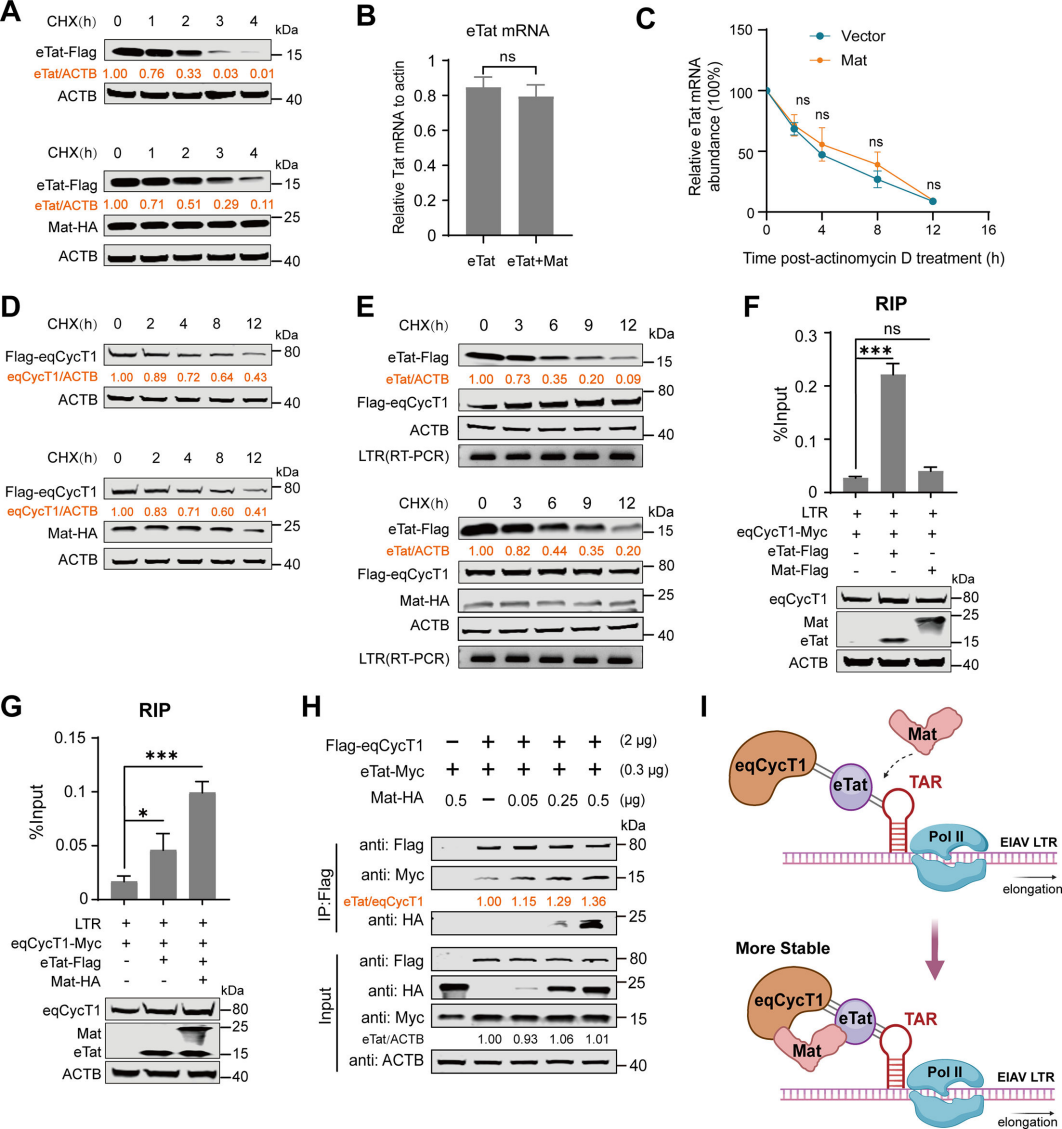
Mat maintained eTat stability and promoted the association of eTat with its transcriptional partners. (**A**) Mat increases eTat stability. HEK293T cells were transfected with eTat-Flag and Mat-HA expression plasmids or an empty vector. At 24 hpt, cells were treated with CHX (50 μg/mL). eTat protein expression was detected by WB. The orange numbers indicate the ratio of eTat to ACTB band intensity, with the value at 0 h set as 1. Data shown are representative of three independent experiments. (**B**) eTat mRNA level measured by RT-qPCR. Data are shown as mean ± SD of three technical replicates from a representative experiment. The experiment was repeated three times independently with similar results. ns, not significant (*P* > 0.05). (**C**) Mat does not affect eTat mRNA stability. HEK293T cells were transfected with eTat-Flag and Mat-HA expression plasmid or an empty vector. At 24 hpt, cells were treated with actinomycin D (10 μg/mL). The eTat mRNA level was detected by RT-qPCR. The eTat mRNA level at 0 h post-treatment was set as 100%, and the remaining mRNA at each time point was expressed as a percentage of the 0-h value. Data are shown as mean ± SD of three technical replicates from a representative experiment. The experiment was repeated three times independently with similar results. ns, not significant (*P* > 0.05). (**D**) Mat does not affect eqCycT1 stability. The orange numbers indicate the ratio of eqCycT1 to ACTB band intensity, with the value at 0 h set as 1. Data shown are representative of three independent experiments. (**E**) The stability of eTat within the transcription complex (eTat/LTR/eqCycT1) in the presence or absence of Mat. TAR RNA of LTR was measured by RT-PCR. The orange numbers indicate the ratio of eTat to ACTB band intensity, with the value at 0 h set as 1. Data shown are representative of three independent experiments. (**F** and **G**) eTat enrichment at the EIAV LTR by RIP with anti-Flag M2 magnetic beads. Results represent percent immunoprecipitated RNA over input after empty vector control background subtraction. Results represent the means ± SD of three replicate wells from a representative experiment. Independent experiments were performed two times. ns, not significant (*P* > 0.05); *P < 0.05; and ***P < 0.001. (**H**) Co-IP analysis of eTat and eqCycT1 with the increasing dose of Mat. HEK293T cells were cotransfected with the indicated plasmids; at 48 hpt, cells were harvested and subjected to Co-IP analysis. The orange numbers indicate the relative eTat to eqCycT1 band intensity in the IP samples, normalized to the value at 0 μg Mat (set as 1). Data shown are representative of three independent experiments. (**I**) Model of Mat’s role in EIAV Tat-mediated LTR transcriptional regulation. Mat stabilizes eTat and enhances the interaction of eTat with eqCycT1 and TAR RNA, thereby promoting EIAV transcriptional elongation.

As an intrinsically disordered protein, HIV-1 Tat undergoes rapid degradation through multiple cellular pathways (20, 23, 24, 33). To determine whether Mat enhances eTat-mediated LTR activity by stabilizing the eTat protein, we performed cycloheximide (CHX) chase assays. HEK293T cells were cotransfected with the eTat plasmid, along with the Mat plasmid or an empty vector control. At 24 hpt, cells were treated with CHX (50 μg/mL) to inhibit protein synthesis, and eTat protein levels were monitored by WB over the subsequent 4 h. The results showed that eTat stability was enhanced in the presence of Mat (Fig. 7A). RT-qPCR analysis confirmed that eTat mRNA levels were not affected by Mat co-expression (Fig. 7B). To determine whether Mat stabilizes eTat by affecting its mRNA stability, we assessed eTat mRNA stability after actinomycin D treatment (10 μg/mL) and found that Mat did not alter eTat mRNA stability (Fig. 7C). These findings indicate that Mat increases eTat stability at the protein level. In parallel CHX chase assays, we found that eqCycT1 stability was unaffected by Mat (Fig. 7D). Given that eTat-mediated LTR activity requires its interaction with TAR RNA and eqCycT1, we cotransfected HEK293T cells with eTat, the pGL3-LTR, eqCycT1, and either Mat or an empty vector and performed CHX chase assays. Within this transcription complex, Mat still enhanced the stability of eTat protein (Fig. 7E). To investigate whether Mat modulates the interaction between eTat and TAR RNA, we performed RNA immunoprecipitation (RIP) assays to assess eTat recruitment to the EIAV TAR RNA in the presence or absence of Mat. RT-qPCR of precipitated RNA confirmed that Mat does not directly bind TAR RNA (Fig. 7F). Notably, Mat enhanced eTat recruitment to TAR RNA compared to controls (Fig. 7G). Furthermore, Mat also enhanced the binding of eTat to eqCycT1 in a dose-dependent manner (Fig. 7H). Together, these results indicate that Mat stabilizes eTat protein and promotes its interactions with both eqCycT1 and TAR RNA, thereby enhancing EIAV LTR activity (Fig. 7I).

## DISCUSSION

Recently, we identified three novel accessory proteins encoded by EIAV. While the functions of two of these proteins (S4 and Grev) have been well elucidated, the role of the third, Mat, remains unclear. S4 is an anti-tetherin protein that promotes EIAV release by downregulating the cell surface expression of the host restriction factor tetherin (34). Grev is a post-transcriptional transactivator that, like Rev, mediates Mat expression (26). In this study, we report that Mat functions as a cofactor of eTat to regulate EIAV replication. Our results demonstrate that inactivation of Mat resulted in attenuated EIAV replication, and this defect was rescued by trans-complementation of Mat. Further investigation showed that Mat enhances eTat-mediated LTR activity in a dose-dependent manner. Confocal microscopy revealed clear nuclear colocalization of Mat and eTat in FDD cells, and their interaction was verified by Co-IP and BiFC assays. Subsequent analysis revealed that Mat maintains eTat stability and promotes its binding to both the TAR and eqCycT1. Notably, Mat interacts with eTat and eqCycT1 but does not interact with TAR RNA. Collectively, these data indicate that Mat functions as an eTat cofactor that enhances the interaction between eTat and the transcriptional complex on the EIAV LTR, thereby facilitating LTR activity and ultimately improving viral replication *in vitro*.

Given that the Mat coding region overlaps with Gag and Env, conventional frameshift or deletion mutations in the mat ORF are not feasible for generating a Mat-inactivated infectious clone, as these would also compromise the functions of Gag and Env. We therefore introduced two synonymous nucleotide mutations near the unique *mat*-specific alternative SA to specifically disrupt the alternative splicing of the mat transcript, thereby abolishing the production of *mat* mRNA (Fig. 1B). Although these mutations are located within the *env* coding sequence, they do not alter the primary structure of the Env protein, nor do they affect Env expression or its ability to mediate viral entry (Fig. 1C). These findings indicate that the attenuated replication of CMV3-8ΔMat is primarily attributable to Mat inactivation, with the possibility of confounding effects from changes in the *env* coding region effectively ruled out. Importantly, this conclusion is supported by successful restoration of viral replication through trans-complementation with Mat. Unfortunately, we were unable to detect Mat at the protein level in the context of the wild-type EIAV infectious clone CMV3-8 (Fig. S1). This is probably due to the low expression of Mat in CMV3-8-transfected cells and the limited sensitivity of the anti-Mat antibody.

In addition to encoding the three common structural proteins (Gag, Pol, and Env), lentiviruses also encode several small viral proteins that fall into two functional categories. One category comprises regulatory proteins, such as Tat and Rev, which specifically regulate viral transcription and the nuclear export of mRNA, respectively. The other category consists of accessory proteins, which are usually dispensable for viral replication *in vitro*, at least in certain cell lines. Representative examples include Vif, Nef, and Vpu from HIV-1, as well as S2 from EIAV (35, 36). Mat is a small protein encoded by EIAV that we recently uncovered. It lacks amino acid sequence homology with small proteins of other lentiviruses. In this study, we found that Mat exhibits nuclear localization and acts as an eTat cofactor to enhance eTat-mediated LTR transcriptional activity in FDD cells (an equine fibroblast cell line). Accordingly, Mat may belong to the category of regulatory proteins. Intriguingly, no canonical nuclear localization signal was predicted in the Mat sequence. Importantly, Mat was predominantly localized at the plasma membrane rather than the nucleus in human cell lines (HeLa and HEK293T) (Fig. S3), which are non-permissive for EIAV. Previous studies indicated that eqCycT1 is indispensable for eTat to exert its transactivation activity, whereas huCycT1 is unable to support this function (13, 18). Here, we found that Mat failed to enhance eTat-mediated LTR activity in HEK293T cells even in the presence of exogenous eqCycT1 (Fig. S4). These findings suggest that the function of Mat may be strictly species-dependent. Accordingly, all functional assays of Mat were carried out in FDD cells in this study, while HEK293T cells were only used to explore the interactions among Mat, eTat, and eqCycT1 due to their high transfection efficiency. Notably, transfection of eMDMs is technically challenging, and high-sensitivity and high-specificity antibodies against Mat are still lacking. Hence, we were unable to characterize the subcellular localization of Mat in equine macrophages, the natural target cells of EIAV. Nevertheless, we confirmed that Mat inactivation attenuates EIAV replication in eMDMs *in vitro*. However, the potential contribution of Mat to EIAV replication and pathogenicity in the host remains undetermined, since most lentiviral accessory proteins are essential for these processes *in vivo*.

It should be noted that when HEK293T and FDD cells were transfected with both the Mat-deficient and wild-type EIAV infectious clones, no notable difference was detected between the two clones in terms of viral expression or virion release. However, when macrophages were infected with viruses derived from these two clones, viral replication of the Mat-deficient virus was moderately reduced relative to that of the wild-type virus. This discrepancy can mainly be attributed to the fact that the EIAV infectious clone used in this study is expressed under the control of the CMV promoter, such that viral expression in transfected cells is independent of eTat-LTR. In contrast, when macrophages are infected with the viruses derived from the EIAV infectious clone, viral replication is driven by eTat-LTR. This speculation is well supported by subsequent Mat trans-complementation assays (Fig. 1I) and luciferase reporter assays (Fig. 2C). Of note, we cannot rule out that disparities in intracellular microenvironments and endogenous host factors among the three cell types may also partially lead to these phenotypic differences.

Previously, Sawaya et al. (21) reported that HIV-1 Vpr interacts with both hTat and huCycT1 to enhance LTR promoter activity, thereby promoting viral replication; notably, this interaction between Vpr and hTat is RNA-dependent. In addition, Joseph et al. (22) found that HIV-1 Nef interacts with hTat to similarly enhance LTR promoter activity and facilitate viral replication (37). Conversely, another study reported that Nef suppresses hTat-mediated transcription by promoting hTat degradation via the proteasome (38). The present study reveals that the EIAV Mat exhibits functional homology with HIV-1 Vpr, as it also enhances LTR promoter activity through binding to eTat and eqCycT1. In contrast to the RNA-dependent nature of the HIV-1 Vpr-Tat interaction, however, the association between EIAV Mat and eTat is RNA-independent. Mechanistically, this enhancement of LTR activity by EIAV Mat is mediated, at least in part, by its ability to increase eTat protein stability and promote the interaction of eTat and its key transcriptional partners (eqCycT1 and the TAR). This dual mechanism of stabilizing eTat and facilitating its assembly into functional transcriptional complexes elucidates the mode of action of EIAV Mat as an eTat cofactor. These observations highlight conserved strategies employed by lentiviral accessory proteins to modulate Tat-dependent viral gene expression, while also underscoring the distinct molecular features that differentiate their modes of action. Regrettably, we were unable to detect the interactions between Mat, eTat, and eqCycT1 in the context of EIAV infection due to the lack of highly sensitive and specific antibodies against these proteins.

In conclusion, we have defined the biological function of EIAV Mat as acting as an eTat cofactor to regulate viral transcription. This finding closes a significant knowledge gap in our understanding of the biology of the recently identified EIAV accessory proteins (Mat, Grev, and S4), as the role of Mat was previously unknown. Nevertheless, Mat inactivation only exerts a moderate influence on EIAV replication *in vitro*. Moreover, the effects of Mat on *in vivo* EIAV replication and pathogenicity remain unclear. In addition, we were unable to characterize the subcellular localization of Mat as well as its interactions with eTat and eqCycT1 in the context of EIAV infection. These limitations need to be elucidated in further research. Taken together, our findings contribute to a broader understanding of lentiviral transcriptional regulation.

## MATERIALS AND METHODS

### Cells

HEK293T and HeLa cells were maintained in Dulbecco’s modified Eagle medium (Sigma-Aldrich, D0819) supplemented with 10% fetal bovine serum (FBS; Alphabio, SA-201-1A) and 1% penicillin-streptomycin (Beyotime, C022). Equine monocyte-derived macrophages (eMDMs) were isolated from fresh heparinized horse whole blood and maintained in RPMI 1640 medium (Sigma-Aldrich, R8758) supplemented with 30% newborn bovine serum (Ausbian, WS500N) and 30% donor horse serum (HyClone, SH30074.03). Fetal donkey dermal (FDD) cells were maintained in minimum essential medium alpha (α-MEM; Gibco, BL306A) supplemented with 10% FBS and 1% penicillin-streptomycin (Beyotime, C022). All cells were cultured at 37°C in 5% CO_2_.

### Plasmids

The Mat-defective EIAV infectious clone (CMV3-8ΔMat) was generated based on CMV3-8 (39), a full-length EIAV infectious clone, by mutating two nucleotides near the SA′ site of *mat* mRNA (T to C at 7849 and T to A at 7852 in the EIAV genome). The pGL3-LTR was generated by inserting EIAV LTR into the pGL3-basic plasmid (40). pRL-CMV, a Renilla luciferase reporter plasmid under the CMV promoter, was used as an internal control for transfection efficiency. pcDNA-Mat-HA, the codon-optimized Mat expression plasmid, was constructed by inserting the codon-optimized Mat sequence into a pcDNA3.1 vector and fusing HA-tags to the C-terminus. pcDNA-Mat-Flag was similarly constructed by inserting the codon-optimized Mat sequence into a pcDNA3.1 vector with a C-terminal Flag tag. The plasmid pcDNA-E1-HA was generated by inserting Exon 1 of the codon-optimized Mat gene sequence into the pcDNA3.1 vector with a C-terminal HA tag. The other five codon-optimized Mat-E1 expression plasmids were synthesized according to the following EIAV strain genome sequences: EIAV_UK_ (GenBank AF016316.1; nucleotides 450–836), EIAV_DE_ (GenBank KM247554.1; nucleotides 114–500), EIAV_Cornwall_ (GenBank MH580898.1; nucleotides 231–617), EIAV_NC-Dagmar_ (GenBank MH820164.1; nucleotides 100–486), and EIAV_Miyazaki_ (GenBank JX003263.1; nucleotides 450–836). For binding domain mapping, VR-GST-E1 was constructed by inserting Exon 1 of the codon-optimized Mat gene into the VR1012 vector with an N-terminal GST tag. Based on this plasmid, a series of VR-GST-E1 truncation mutants were generated. For eTat expression, three constructs with different C-terminal tags were generated based on the VR1012 vector: VR-eTat-Flag, VR-eTat-HA, and VR-eTat-Myc. For the dual-luciferase reporter assay, the pCAGGS-Flag-eTat was constructed by inserting the eTat gene sequence into a pCAGGS vector and fusing Flag tags to the N-terminus. For eqCycT1 expression, three plasmids were constructed: pCAGGS-Flag-eqCycT1, encoding eqCycT1 with an N-terminal Flag tag in the pCAGGS vector; and VR-eqCycT1-Myc and VR-eqCycT1-HA, encoding eqCycT1 with a C-terminal Myc or HA tag, respectively, in the VR1012 vector. For BiFC assays, pcDNA-Mat-VN, pcDNA-Mat-VC, pcDNA-eTat-VN, and pcDNA-eTat-VC were constructed by fusing the VN (residues 1–173) or VC (residues 174–239) fragment of Venus to the C-terminus of Mat or eTat. For Gag-Pol expression, pGP-RRE was constructed by inserting the *gag-pol-RRE* gene into pEGFP-N1, as previously described (25). The Rev expression plasmid, VR-Rev-Flag, was constructed by inserting the EIAV Rev gene sequence into a VR1012 vector and fusing Flag-tags to the C-terminus. pcDNA-Env, a wild-type Env expression plasmid, was constructed previously (41). pcDNA-Env/SA was derived from pcDNA-Env, with two nucleotide mutations corresponding to the splicing site of Mat. All constructed plasmids were verified by sequencing and are summarized in Tables S1 and S2.

### Transfection

HEK293T and HeLa cells were transfected with the indicated plasmids using Polyethylenimine HCl MAX (MW 40,000; Polysciences, 24765-1). FDD cells were transfected using Lipofectamine LTX with PLUS Reagent (Invitrogen, 15338100) according to the manufacturer’s protocol. All transfections were performed at least three times.

### Antibodies and reagents

The following antibodies were used in this study: mouse anti-Flag monoclonal antibody (Sigma-Aldrich, F1804, 1:5,000); rabbit anti-HA polyclonal antibody (Sigma-Aldrich, H6908, 1:5,000); mouse anti-Myc monoclonal antibody (Abmart, M20002, 1:5,000); rabbit anti-beta actin monoclonal antibody (Abclonal, AC026, 1:5,000); and rabbit anti-GST polyclonal antibody (Proteintech, 10000-0-AP, 1:5,000). A mouse monoclonal antibody against p26 (9H8) (1:4,000), which recognizes the capsid protein of EIAV, was generated in our laboratory (42). To detect Mat expression in CMV3-8, a mouse anti-Mat monoclonal antibody (1G3) was used (1:500). This antibody was produced by immunization of mice with a KLH carrier protein containing a polypeptide corresponding to the C-terminal 15 amino acids (ENAKSSYISCNNASI) of Mat (25). Biotin-conjugated Affinipure goat anti-mouse IgG (H + L) (Proteintech, SA00004-1, 1:1,000) and IRDye 800CW-conjugated streptavidin (LI-COR, 925-32230, 1:5,000) were used as secondary detection reagents following 1G3.

For all other WB experiments, the following secondary antibodies were used: DyLight 800-labeled goat anti-mouse IgG (H + L) (KPL, 072-06-15-16, 1:10,000) and DyLight 680-labeled goat anti-rabbit IgG (H + L) (Invitrogen, 35568, 1:5,000).

Cycloheximide (HY12320) and actinomycin D (HY-17559) were purchased from MedChemExpress.

### Reverse transcription PCR

To identify the defect of *mat* in CMV3-8, HEK293T cells were transfected with either the CMV3-8 or CMV3-8ΔMat expression plasmid. At 48 hpt, total RNA was extracted from the collected cells using the TRIzol reagent. The RNA was then reverse-transcribed into cDNA using the PrimeScript Fast RT reagent Kit with gDNA eraser (Takara, RR047B) according to the manufacturer’s instructions. Target gene fragments were amplified by PCR using gene-specific primers. For the *mat* gene, a nested PCR approach was employed. The first round of amplification used the outer primer pair p555 (forward: 5′-TCAAAAGCTAACTAATGGTAAC-3′) and p7878 (reverse: 5′-ATTGAGGCATTGTTACATGAGA-3′). The resulting product served as the template for the second round, which used the inner primer pair p643 (forward: 5′-CAATTAAGGGACGTCATTCCAT-3′) and p7855 (reverse: 5′-GTAGCTGGATTTAGCATTTTCC-3′). The *gag* and *env* gene fragments were amplified using standard PCR with the following primers: Gag-F (forward: 5′-GCAAATACTACCAAAAAGC-3′) and Gag-R (reverse: 5′-AGTGTCTGTCAGGAATTTAG-3′) for *gag*; Env-F (forward: 5′-TATCATGCATACCTGGC-3′) and Env-R (reverse: 5′-CTGGGTTACATGAGGTTATGAGAG-3′) for *env*. The amplification conditions for the *mat* gene were as follows, with identical parameters for both rounds of nested PCR: 2 min at 98°C; 35 cycles of 30 s at 98°C, 30 s at 52°C, and 30 s at 68°C; and a final extension step of 10 min at 68°C. The amplification conditions for the *gag* and *env* genes were as follows: 2 min at 98°C; 35 cycles of 30 s at 98°C, 30 s at 58°C, and 60 s at 68°C; and a final extension step of 10 min at 68°C. All PCR products were resolved by agarose gel electrophoresis, and specific bands were excised and purified using a gel extraction kit (Vazyme, DC301-01).

For EIAV LTR detection in the CHX chase assay, the primer pair U5-F (forward: 5′-ATTTGGTCGCTCAGATTCTGC-3′) and Fluc-R (reverse: 5′-CACGTTCATTATAAATGTCG-3′) was used. The amplification conditions were as follows: 2 min at 98°C; 35 cycles of 30 s at 98°C, 30 s at 56°C, and 30 s at 68°C; and a final extension step of 10 min at 68°C.

### Dual-luciferase reporter assay

FDD cells were cultured in 24-well plates overnight before transfection. The cells were transfected with 0.2 μg of pCAGGS-Flag-eTat, 0.3 μg of pGL3-LTR-Luc, and different amounts of pcDNA-Mat-HA plasmid. An empty pcDNA3.1 vector was added to normalize the amount of DNA transfected in each well. In addition, the Renilla luciferase construct pRL-CMV (0.1 μg) was cotransfected as a control to normalize the transfection efficiency. At 24 hpt, cells were lysed, and the luciferase activity was measured using a dual-luciferase reporter assay system (Promega, USA) according to the manufacturer’s instructions.

### RNA stability assay

HEK293T or FDD cells were transfected with the indicated plasmids. At 24 hpt, cells were treated with actinomycin D (10 μg/mL) to block transcription. Cells were harvested at 0, 2, 4, 8, and 12 h post-treatment. At each time point, total RNA was extracted using a Simply P Total RNA Extraction Kit (BioFlux, BSC52M1) and reverse-transcribed using the PrimeScript RT Reagent Kit with gDNA Eraser (Takara, RR047B) according to the manufacturer’s instructions. The expression levels of eTat mRNA (or Fluc mRNA) and the housekeeping control ACTB mRNA were quantified by SYBR Green (Takara, RR430A)-based quantitative real-time PCR on a QuantStudio 5 system. The following primer pairs were used: eTat-F (5′-TGGCCACAACACAGGAAGAC-3′) and eTat-R (5′-GCGAGCTGTCAAGGTAGTCAA-3′) for eTat detection; ACTB-F (5′-CATCTGCTGGAAGGTGGACAA-3′) and ACTB-R (5′-CGACATCCGTAAGGACCTGTA-3′) for ACTB detection; qFluc-F (5′-AGAGATACGCCCTGGTTCCT-3′) and qFluc-R (5′-TCCGATAAATAACGCGCCCA-3′) for Fluc mRNA detection. Relative mRNA levels were calculated using the 2^(−ΔΔCt)^ method, normalized to ACTB as an internal control. The mRNA level at 0 h post-treatment was set as 100%, and the remaining mRNA at each time point was expressed as a percentage of the 0-h value. The cycling conditions were as follows: 95°C for 2 min; 40 cycles of 95°C for 30 s and 58°C for 30 s, followed by a melt curve stage (95°C for 1 min, 55°C for 30 s, and 95°C for 30 s).

### Western blotting

Cells were lysed using ice-cold lysis buffer (50 mM Tris-HCl [pH 7.5], 50 mM NaCl, 5 mM EDTA, and 1% Triton X-100) and centrifuged at 13,000 × *g* and 4°C for 10 min. The proteins were separated using SDS-PAGE and then transferred to nitrocellulose membranes (Merck-Millipore, HATF00010). Following this, the membranes were blocked for 2 h with 5% skim milk (Biosharp, BS102-100g) in phosphate-buffered saline (PBS). The blots were probed with primary antibodies for 2 h and then incubated with secondary antibodies for 1 h. The membranes were analyzed on the LI-COR Odyssey Imaging System (LI-COR, USA).

### Co-IP assay

HEK293T or FDD cells were cultured in 6 cm dishes. At 48 hpt, cells were harvested and lysed using ice-cold lysis buffer (50 mM Tris-HCl [pH 7.5], 50 mM NaCl, 5 mM EDTA, and 1% Triton X-100). The lysates were centrifuged at 13,000 × *g* and 4°C for 10 min to remove cell debris. Then, the cellular extracts were incubated with anti-Flag M2 magnetic beads (Sigma-Aldrich, M8823) at 4°C overnight. Following the incubation, the beads were washed three times with 1× PBS, and then bound proteins were eluted by mixing and heating the beads in sample loading buffer for 5 min at 98°C. The samples were subjected to SDS-PAGE followed by immunoblotting analysis using corresponding antibodies.

### Confocal microscopy

FDD, HeLa, and HEK293T cells were transfected with the indicated plasmids and harvested at 24 hpt. Cells were fixed with 4% paraformaldehyde (Beyotime, P0099-500 mL) for 30 min and then permeabilized with 0.1% Triton X-100 for 15 min. After being blocked with 5% skim milk for 1 h, samples were subjected to an immunofluorescence assay. Mouse anti-Flag antibody (Sigma-Aldrich, F1804) (1:200) and rabbit anti-HA antibody (Sigma-Aldrich, F7425) (1:200) were added in the blocking buffer, and cells were incubated for 2 h, followed by staining using Alexa Fluor 488-conjugated goat anti-rabbit antibody (Invitrogen, A32731, 1:500) and Alexa Fluor 647-conjugated goat anti-mouse antibody (Invitrogen, A32738, 1:500) for 1 h. Cells were washed with 1× PBS three times after each step. Finally, the samples were observed and imaged on a confocal microscope (ZEISS LSM800).

### Replication of the Mat-deficient EIAV in eMDMs

To detect the replication capacity of Mat-deficient EIAV in eMDMs, eMDMs were infected with equivalent titers of EIAV_CMV3-8_ and EIAV_CMV3-8ΔMat_ at an MOI of 1 or 0.1. After 2 h of infection, the supernatant was replaced with fresh culture medium. Equal volumes of cell culture supernatants were collected at indicated time points. Viral RNAs in the supernatant samples were extracted using the QIAamp Viral RNA Mini Kit (Qiagen, DP315-R) and then subjected to reverse transcription using the PrimeScript RT reagent Kit with gDNA Eraser (Takara, RR047B) according to the manufacturer’s instructions. The level of EIAV replication was determined by qPCR to detect the genomic RNA using *gag* gene-specific primers: Gag-F (forward: 5′-CGAATGCCAAATCCTCCATTAG-3′) and Gag-R (reverse: 5′-CTGATCAAAAGCAGGTTCCATCT-3′), as described previously (43).

### Production of pseudotyped HIV encoding Mat

An HIV-1-based reporter virus system was constructed using three plasmids: pSIN4-EF1a-IRES-GFP, psPAX2, and pMD2.G. The pSIN4-EF1a-IRES-GFP transfer vector was derived from the commercial plasmid pSIN4-EF1a-LMO2-IRES-Puro (Addgene #61064) by deleting the LMO2 gene and replacing the puromycin resistance gene with GFP. psPAX2 is an HIV-1 packaging helper plasmid, and pMD2.G expresses the vesicular stomatitis virus glycoprotein G. This system was used to overexpress Mat in eMDMs. The recombinant vector pSIN4-EF1a-Mat-Flag-IRES-GFP, which expresses Mat protein with a C-terminal Flag tag, was constructed by inserting the EIAV Mat ORF sequence between the EF1α promoter and the IRES-GFP element using overlap extension PCR.

To generate pseudotyped HIV-1 particles containing Mat-Flag or lacking Mat-Flag (used as a negative control), HEK293T cells were transfected with pSIN4-EF1a-Mat-Flag-IRES-GFP or pSIN4-EF1a-IRES-GFP, together with psPAX2 and pMD2.G, using Lipofectamine LTX with PLUS Reagent (Invitrogen, 15338100). At 48 hpt, the culture supernatants were collected, centrifuged at 300 × *g* for 10 min, and filtered through a 0.45-μm filter to remove cell debris. The supernatants were then centrifuged at 20,000 × *g* at 4°C for 2 h to concentrate the pseudotyped HIV-1 particles. Viral pellets were resuspended in Opti-MEM and quantified using a reverse transcriptase activity kit (Roche, Basel, Switzerland) before being used to infect eMDMs.

### Preparation of EIAV reporter virus expressing luciferase

HEK293T cells were seeded in 6-well plates and cultured overnight. Cells were cotransfected with pUKGag-Pol (1 μg), pONY8.1 (1 μg), and pcDNA-Env or pcDNA-Env/SA (0.31 μg) using the PEI transfection reagent to generate a luciferase-expressing EIAV reporter virus. At 48 hpt, the luciferase reporter virus was quantified using a reverse transcriptase activity kit (Roche, Basel, Switzerland) and subsequently used to infect HEK293T/ELR1 cells (a HEK293T cell line stably expressing the EIAV receptor equine lentivirus receptor 1 [ELR1]) (44).

### Quantification of EIAV viral titers by RT-qPCR

In viral infection assays, RT-qPCR was performed to quantify the genomic copy number of inoculated viruses. HEK293T cells were transfected with CMV3-8 or CMV3-8ΔMat. At 48 hpt, the supernatants were collected and centrifuged at 8,000 × *g* for 10 min to remove cell debris. Viral RNA was subsequently extracted and analyzed via RT-qPCR using the gag-specific primers Gag-F and Gag-R as described above. Since each lentiviral particle contains two copies of the viral genome, the multiplicity of infection was calculated by dividing half of the total viral genomic copy number by the number of infected cells.

### RNA-immunoprecipitation assay

HEK293T cells were cultured in 10 cm dishes and transfected with the indicated plasmids. At 24 hpt, cells were harvested, washed three times with 1× PBS, and lysed in 1 mL of freshly prepared lysis buffer (50 mM Tris-HCl [pH 7.5], 50 mM NaCl, 5 mM EDTA, and 1% Triton X-100) supplemented with 0.2 mM protease inhibitor and 100 U/mL RNase inhibitor. A 100 µL aliquot of the total lysate was saved as the input control. The remainder of the lysate was incubated with anti-Flag M2 magnetic beads (Sigma-Aldrich, M8823) overnight at 4°C with gentle rotation. After three washes with 1× PBS, RNA was extracted from both the immunoprecipitated beads and the input samples using TRIzol reagent. The purified RNA was then reverse transcribed into cDNA (Takara, RR047B) and analyzed by qPCR to quantify associated RNAs. The primer pair used for TAR RNA detection was as follows: qFluc-F (forward: 5′-AGAGATACGCCCTGGTTCCT-3′) and qFluc-R (reverse: 5′-TCCGATAAATAACGCGCCCA-3′). The cycling conditions were as follows: 95°C for 2 min; 40 cycles of 95°C for 30 s and 58°C for 30 s, followed by a melt curve stage (95°C for 1 min, 55°C for 30 s, and 95°C for 30 s).

### Statistical analysis

Band intensities were quantified using Image Studio software (LI-COR). All statistical analysis was performed using GraphPad Prism 5 (GraphPad Software). Statistical parameters are reported in the figures and figure legends. ns, not significant (*P* > 0.05); *P < 0.05; **P < 0.01; ***P < 0.001; and ****P < 0.0001.

## Data Availability

The reference sequences used in this study are available from GenBank under the following accession numbers: GU385361.1, AF016316.1, KM247554.1, MH580898.1, MH820164.1, and JX003263.1. The *mat* gene sequence is available in the GenBank database under accession number OM417133.

## SUPPLEMENTAL MATERIAL

Supplemental material jvi.00828-26-s0001.pdf Fig. S1 to S4; Tables S1 and S2.

ASM does not own the copyrights to Supplemental Material that may be linked to, or accessed through, an article. The authors have granted ASM a non-exclusive, world-wide license to publish the Supplemental Material files. Please contact the corresponding author directly for reuse.
