# Supplementary material for: Equine infectious anemia virus Mat protein serves as a Tat cofactor to facilitate viral transcription
Source: J Virol. 2026 Jul 27;100(8):e00828-26. doi: 10.1128/jvi.00828-26 (PMC13483383; doi:10.1128/jvi.00828-26)
Supplement: Supplementary file 1 [file jvi.00828-26-s0001.pdf]

Equine Infectious Anemia Virus Mat protein serves as a Tat cofactor to facilitate viral transcription

Xiangmin Zhang[1], Weiwei Ma[1], Xiaohua Ma[1], Xing Guo[1], Weiguo Zhang[1], Xue-Feng Wang[1*], Xiaojun Wang[1,2,3*]

[1] State Key Laboratory of Animal Disease Control and Prevention, Harbin Veterinary Research Institute of Chinese Academy of Agricultural Sciences, Harbin, China

[2]Institute of Western Agriculture, the Chinese Academy of Agricultural sciences, Changji 831100, PR China

[3]China-Kazakhstan Joint Laboratory for Herbivorous Animal Disease Research, Heilongjiang Province， Harbin Veterinary Research Institute, Chinese Academy of Agricultural Sciences, Harbin 150069, China

[*]Corresponding Author.

E-mail: wangxiaojun@caas.cn (XW)； wangxuefeng@caas.cn (X-F.W)

Supplemental Material

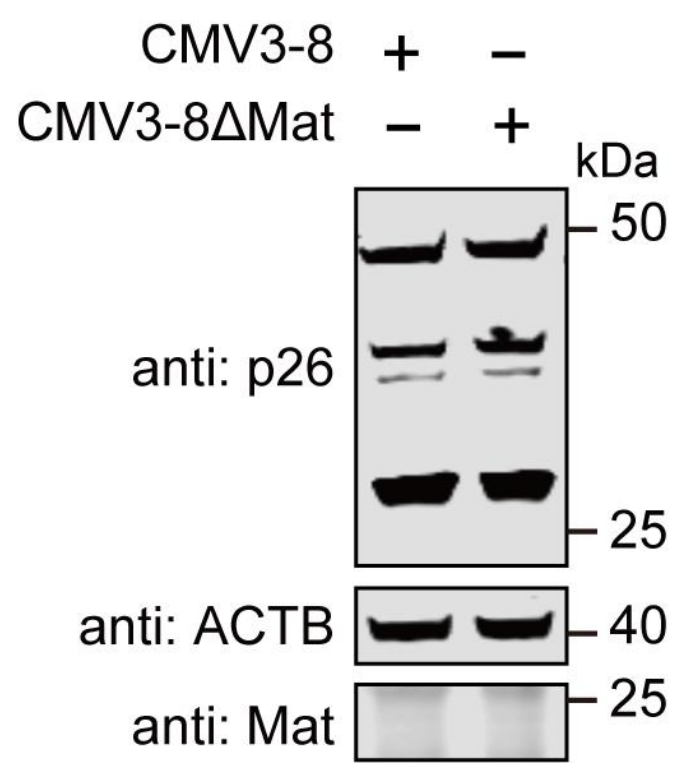

Figure S1. Mat expression in HEK293T cells transfected with infectious clones CMV3-8 and CMV3-8ΔMat. HEK293T cells were transfected with CMV3-8 and CMV3-8ΔMat. At 48 hpt, cells were harvested and analyzed by WB. Mat was detected using a mouse anti-Mat mAb (1G3, 1:500) as the primary antibody, biotin-conjugated goat anti-mouse IgG (Proteintech, SA00004-1, 1:1000) as the secondary antibody, and IRDye 800CW streptavidin (LI-COR, 925-32230, 1:5000) as the tertiary reagent.

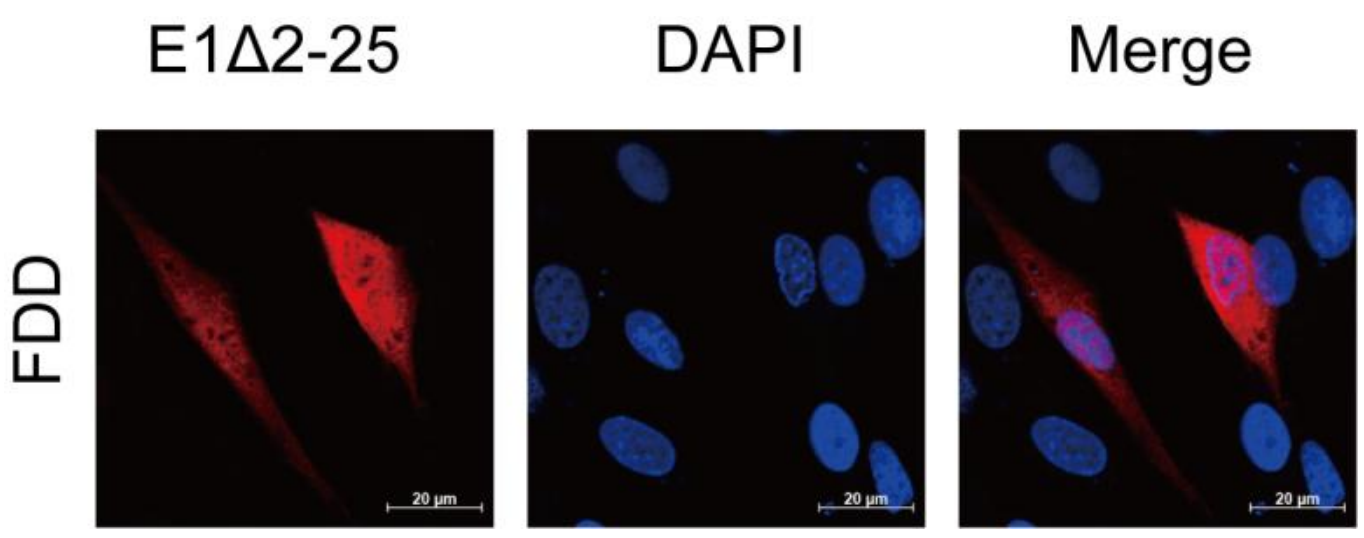

Figure S2. Subcellular localization of E1Δ2-25-HA. FDD cells were transfected with E1Δ2-25-HA expression plasmid. At 24 hpt, cells were fixed and stained with an anti-HA antibody (red) to detect the E1 protein, and with DAPI (blue) to label the nucleus. Scale bar: 20 μm. Representative images of 20 cells are shown.

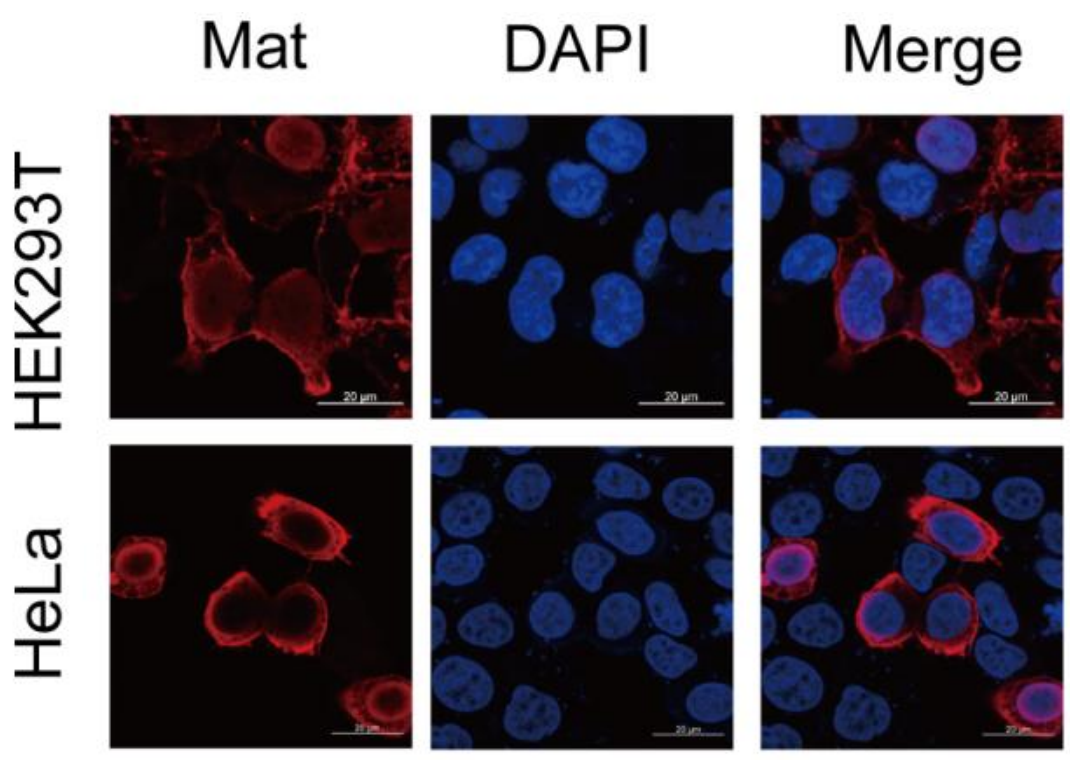

Figure S3. Subcellular localization of ectopically expressed Mat protein in HEK293T and HeLa cells. Mat-HA expression plasmid was transfected into the indicated cells, at 24 hpt, cells were fixed, permeabilized, and immunostained with rabbit anti-HA (red) followed by Alexa Fluor 647-conjugated goat anti-rabbit antibodies. Nuclei were counterstained with DAPI (blue). Then observed using confocal microscopy. Scale bar: 20 μm. Representative images of 20 cells are shown.

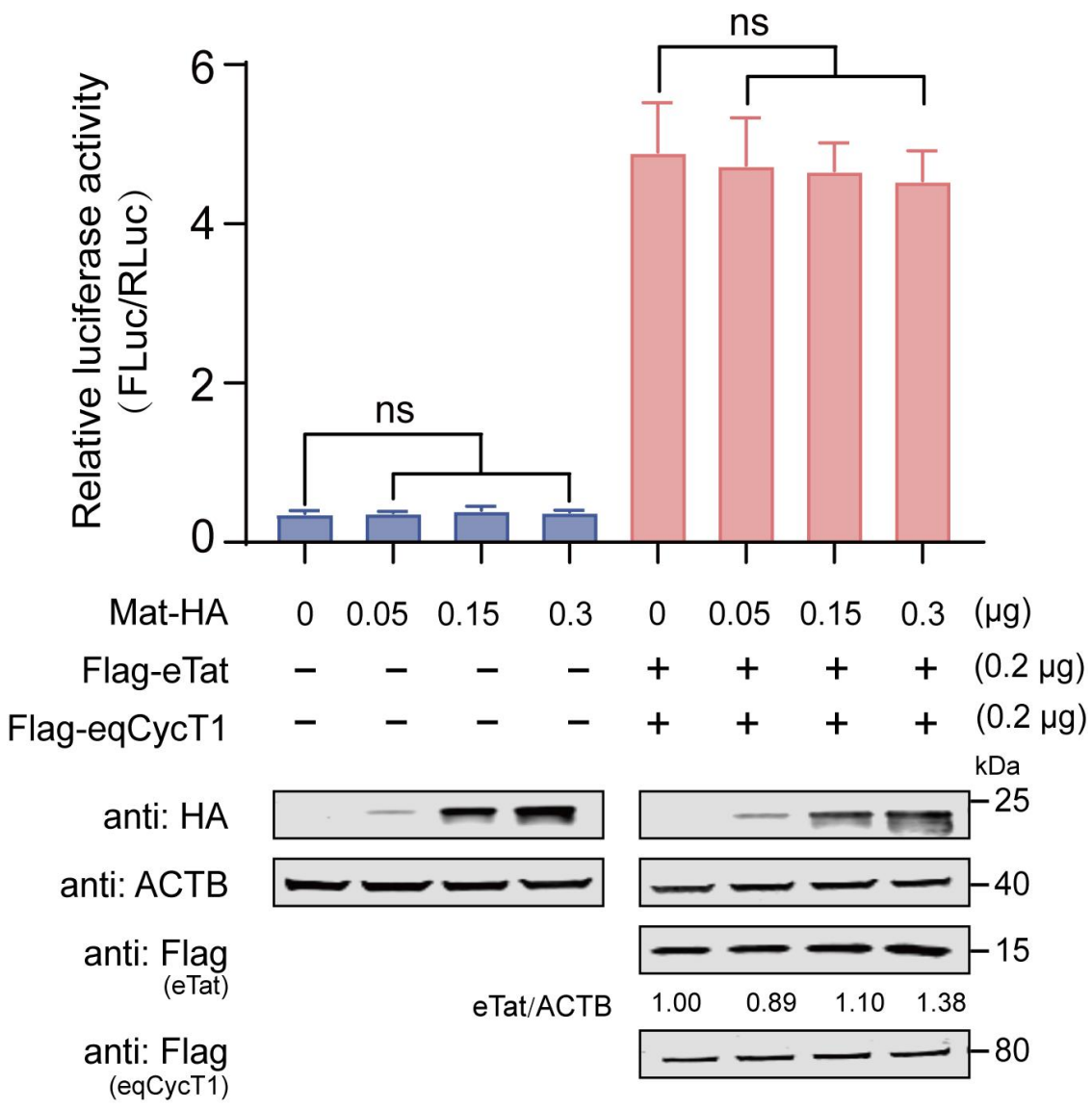

Figure S4. Mat failed to enhance eTat-mediated LTR activity in HEK293T cells. HEK293T cells were cotransfected with pGL3-LTR and the indicated plasmids. The Renilla luciferase expression plasmid pRL-CMV was cotransfected to normalize transfection efficiency. An empty vector pcDNA3.1(+) was added to normalize the DNA amounts of transfection. The relative luciferase activities were measured to assess the EIAV LTR transcriptional activity. Results represent the means ± SD of three replicate wells from a representative experiment. Independent experiments were performed three times. ns, not significant ($P > 0.05$).

Table S1. EIAV infectious clones and Mat expression constructs

Construct name	Vector	Insert gene	Tag	Description	Experimental purpose
CMV3-8	pEGFP-N1	*EIAV*-FDDV full-length genome	None	Full-length EIAV-FDDV infectious clone	Viral replication experiments
CMV3-8 ∆ Mat	pEGFP-N1	*EIAV*-FDDV full-length genome (lacking *mat*)	None	*mat*-defective EIAV-FDDV infectious clone	Viral replication experiments
pHIV-Mat	pSIN4-EF1a-IRES-GFP	*EIAV*-FDDV *mat*	C-terminal Flag	HIV-1-based transfer vector	Mat expression in eMDMs
pcDNA-Mat-HA	pcDNA3.1	*EIAV*-FDDV *mat*	C-terminal HA	Full-length Mat from *EIAV*-FDDV	Mat expression
pcDNA-Mat-Flag	pcDNA3.1	*EIAV*-FDDV *mat*	C-terminal Flag	Full-length Mat from *EIAV*-FDDV	Mat expression
pcDNA-Mat-HA-VC	pcDNA3.1	*EIAV*-FDDV *mat*	C-terminal HA	Mat from *EIAV*-FDDV fused C-terminal Venus	BiFC assay
pcDNA-Mat-HA-VN	pcDNA3.1	*EIAV*-FDDV *mat*	C-terminal HA	Mat from *EIAV*-FDDV fused N-terminal Venus	BiFC assay
pcDNA-E1-HA	pcDNA3.1	*EIAV*-FDDV *mat* exon1	C-terminal HA	Mat Exon1 from *EIAV*-FDDV	Mat Exon1 expression
pcDNA-E1-UK-HA	pcDNA3.1	*EIAV*-UK *mat* exon1	C-terminal HA	Mat Exon1 from *EIAV*-UK	Dual-luciferase reporter assay
pcDNA-E1-DE-HA	pcDNA3.1	*EIAV*-DE *mat* exon1	C-terminal HA	Mat Exon1 from *EIAV*-DE	Dual-luciferase reporter assay
pcDNA-E1-Cornwall-HA	pcDNA3.1	*EIAV*-Cornwall *mat* exon1	C-terminal HA	Mat Exon1 from *EIAV*-Cornwall	Dual-luciferase reporter assay
pcDNA-E1-Dagmar-HA	pcDNA3.1	*EIAV*-Dagmar *mat* exon1	C-terminal HA	Mat Exon1 from *EIAV*-Dagmar	Dual-luciferase reporter assay
pcDNA-E1-Miyazaki-HA	pcDNA3.1	*EIAV*-Miyazaki *mat* exon1	C-terminal HA	Mat Exon1 from *EIAV*-Miyazaki	Dual-luciferase reporter assay
VR-GST-E1	VR1012	*EIAV*-FDDV *mat* exon1	N-terminal GST	Mat Exon1 from *EIAV*-FDDV	Mapping binding domains
VR-GST-E1 (Truncations)	VR1012	*EIAV*-FDDV *mat* exon1	N-terminal GST	Truncation series of Mat Exon1from *EIAV*-FDDV	Mapping binding domains

Note: BiFC=Bimolecular fluorescence complementation；eMDMs = equine monocyte-derived macrophages

Table S2. EIAV Tat, eqCycT1 and reporter constructs

Construct name	Vector	Insert gene	Tag	Description	Experimental purpose
VR-eTat-Flag	VR1012	*EIAV*-FDDV *tat*	C-terminal Flag	*EIAV*-FDDV Tat protein	*EIAV*-FDDV Tat expression
VR-eTat-HA	VR1012	*EIAV*-FDDV *tat*	C-terminal HA	*EIAV*-FDDV Tat protein	*EIAV*-FDDV Tat expression
VR-eTat-Myc	VR1012	*EIAV*-FDDV *tat*	C-terminal Myc	*EIAV*-FDDV Tat protein	*EIAV*-FDDV Tat expression
pCAGGS-Flag-eTat	pCAGGS	*EIAV*-FDDV *tat*	N-terminal Flag	*EIAV*-FDDV Tat protein	Dual-luciferase reporter assay
pcDNA-eTat-Flag-VN	pcDNA3.1	*EIAV*-FDDV *tat*	N-terminal Flag	Tat from *EIAV*-FDDV fused N-terminal Venus	BiFC assay
pcDNA-eTat-Flag-VC	pcDNA3.1	*EIAV*-FDDV *tat*	N-terminal Flag	Tat from *EIAV*-FDDV fused C-terminal Venus	BiFC assay
pCAGGS-Flag-eqCycT1	pCAGGS	*Equus caballus CCNT1*	N-terminal Flag	Equine CycT1 protein (eqCycT1)	Equine CycT1 expression
VR-eqCycT1-HA	VR1012	*Equus caballus CCNT1*	C-terminal HA	Equine CycT1 protein (eqCycT1)	Equine CycT1 expression
VR-eqCycT1-Myc	VR1012	*Equus caballus CCNT1*	C-terminal Myc	Equine CycT1 protein (eqCycT1)	Equine CycT1 expression
pGP-RRE	pEGFP-N1	*EIAV*-FDDV *gag-pol-RRE*	None	*EIAV*-FDDV Gag-Pol polyprotein expression	*EIAV*-FDDV Gag-Pol expression
VR-Rev-Flag	VR1012	*EIAV*-FDDV *rev*	C-terminal Flag	*EIAV*-FDDV Gag-Pol polyprotein expression	*EIAV*-FDDV Rev expression
pcDNA-Env	pcDNA3.1	*EIAV*-FDDV *env*	None	*EIAV*-FDDV Env protein (wild-type)	Preparation of EIAV reporter virus expressing luciferase
PcDNA-Env/SA	pcDNA3.1	*EIAV*-FDDV *env* (mutation)	None	*EIAV*-FDDV Env protein with SA mutation	Preparation of EIAV reporter virus expressing luciferase
pGL3-LTR-Fluc	pGL3-basic	*EIAV*-FDDV LTR	None	Firefly luciferase reporter plasmid driven by *EIAV*-FDDV LTR promoter	Dual-luciferase reporter assay
pRL-CMV	pRL-CMV	None	None	Renilla luciferase reporter plasmid under CMV promoter (internal control)	Dual-luciferase reporter assay

Note: BiFC=Bimolecular fluorescence complementation
